# Les manifestations orthopédiques de la neurofibromatose de type 1 ou maladie de Recklinghausen (VRH)

**DOI:** 10.11604/pamj.2015.21.316.7282

**Published:** 2015-08-28

**Authors:** Mouna Sghir, Wafa Said

**Affiliations:** 1 Unité de Médecine Physique, CHU Taher Sfar Mahdia, Tunisie

**Keywords:** Neurofibromatose de type 1, manifestations orthopédiques, enfant, Neurofibromatosis type 1, orthopedic manifestations, child

## Image en medicine

Les manifestations orthopédiques de la maladie de VRH sont fréquentes, et ce quels que soient l’âge du malade et la nature des autres manifestations de la maladie. Nous rapportons le cas d'un jeune âgé de 9 ans issu d'un père porteur de VRH, adressé pour une déformation du tronc (A), l'examen clinique trouve des tâches café au lait (B), un dos creux (C) et une gibbosité dorsale gauche (D), un trouble rotationnel des membres inférieurs (exagération de l'antéversion fémorale) et des pieds creux au podoscope (E). L'examen neurologique montre une irritation pyramidale. Le bilan radiologique a conclu à la présence d'une scoliose double majeure de 40° en dorsale gauche et 36° en lombaire droite et un scalloping du mur postérieur, absence de lacune osseuse au niveau du crane, et pas de pseudarthrose aux membres inférieurs. Le diagnostic de VRH est confirmé sur l'existence de cas dans la famille (père et sœur) et des signes cutanés associés aux signes rachidiens. Une IRM médullaire demandée à la recherche de lésion tumorale nerveuse est revenue normale. Le patient a bénéficié d'une orthèse du tronc, de paire de semelle orthopédique et d'un protocole de rééducation adapté.

**Figure 1:**
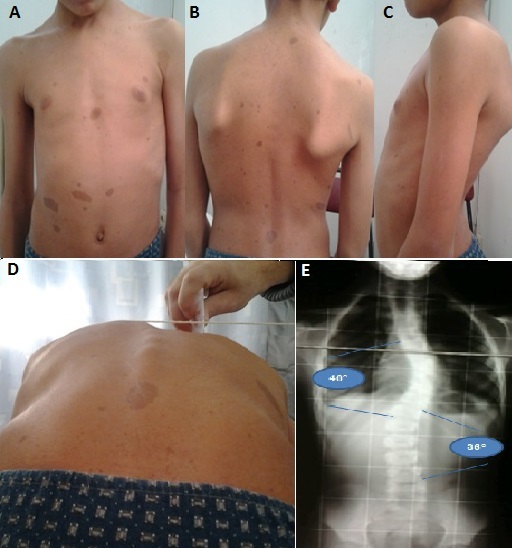
A): Tronc rétro-projeté; B): taches café au lait, déséquilibre des épaules; C): dos plat, décollement des omoplates; D): gibbosité dorsale gauche; E): scoliose dorsale 40° lombaire 30°

